# Physicochemical evaluation, GC–MS-based phytoconstituent analysis and network pharmacology study of Guduchi Arka: an ayurvedic hydro-distillate

**DOI:** 10.1186/s40643-026-01127-z

**Published:** 2026-10-09

**Authors:** Deepa Sharma, Ganesh Dane, Smriti Tandon, Chandrashekhar Jagtap, Vijay Kumar, Suman Mondal, Akanksha Thakur, Vaibhav Charde, Hemant Rawat, Ravi Nahar, Sanjeev Kumar Lale, B. S. Sharma, Shruti Khanduri, T. Maheshwar, N. Srikanth, Rabinarayan Acharya

**Affiliations:** 1 Central Ayurveda Research Institute, Jhansi, Uttar Pradesh 284003 India; 2 All India Institute of Ayurveda, New Delhi, 110076 India; 3 Indian Bureau of Mines (IBM), Nagpur, India; 4 Central Council for Research in Ayurvedic Sciences, Ministry of Ayush, New Delhi, 110058 India

**Keywords:** Amrita, Arka, Guduchi, Hydro-distillate, In-silico study, Network pharmacology

## Abstract

Guduchi Arka is a classical Ayurvedic hydro-distillate formulation with quick absorption and healing properties, prepared from *Tinospora cordifolia* (Willd.) Miers. Despite its long history of traditional use, the scientific community still lacks a complete set of quality parameters for its chemical composition and its molecular mechanisms. To overcome this problem, laboratory analysis and computer modelling parameters were used to investigate the quality, safety, and effectiveness of the formulation in treating fever and cold. Guduchi samples were obtained from three locations in India (Jhansi, Bengaluru, and Itanagar), and the raw materials were analysed to verify compliance with API standards. Authenticated Guduchi samples were processed arka preparation by using a standardised hydro-distillation method, and the resulting arka was assessed by physicochemical analysis, with a pH of 6.15–6.95, a specific gravity near 1.00, and an essential oil content of approximately 0.236%. Raw drugs and Arka preparations complied with standard API safety standards, as no pathogenic organisms were detected, heavy metals, pesticides, and aflatoxins were undetectable, and microbial content was below the standard API limit. GC–MS analysis of the arka formulation revealed a consistent and rich array of bioactive compounds, including 1-Eicosanol, α-Campholenal, o-toluric acid, Anethole, Estragole, Embelin, Nicolaioidesin A, (-)-Globulol, Paliclavine, and Chamazulene. To assess its therapeutic significance, 20 phytoconstituents of the Guduchi arka formulation were screened using network pharmacology and molecular docking. These compounds showed 248 overlapping targets with genes associated with fever and respiratory infections. The key regulatory proteins HSP90AA1, EGFR, and HSP90AB1 emerged as central nodes in the protein–protein interaction networks. To better validate the molecular docking study, robust biological interactions were observed: Nicolaiodesin A interacted effectively with EGFR (score: − 7.2 kcal/mol), whereas Embelin interacted effectively with HSP90AA1 (score: − 6.6 kcal/mol). Finally, this study demonstrates that Guduchi Arka is a highly standardised and safe formulation that retains all its healing phytochemicals during the distillation process. This study elucidates the mode of action of Guduchi Arka on immunity and inflammation, providing a solid scientific basis for its traditional use and future applications in herbal medicine.

**Graphical abstract:**

**Figure:**
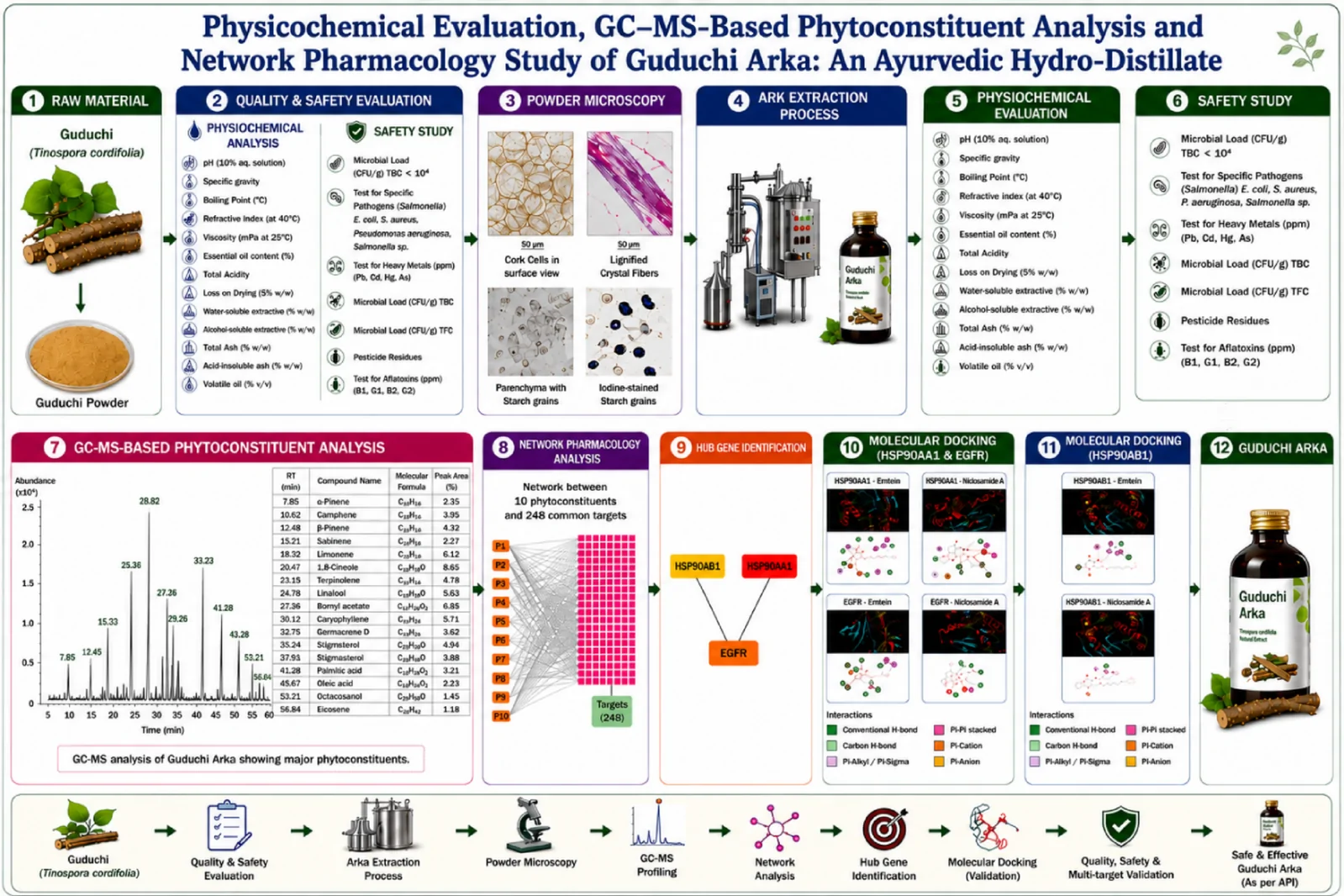

## Introduction

Among the most common conditions seen in clinical practice are fever and upper respiratory tract infections (URTIs), including the common cold. Individuals of all ages and from all geographic regions suffer from these ailments. In addition to causing physical discomfort, such as fever, sore throat, nasal congestion, and exhaustion, these disorders also reduce productivity and quality of life, particularly in susceptible groups, including children, the elderly, and people with impaired immune systems. Antipyretics, antihistamines, and antibiotics are commonly used medications, however conventional therapy often has drawbacks, including antibiotic resistance, side effects (Chow et al. 2012), and a lack of comprehensive immunological support. (Fendrick et al. 2003; Heikkinen and Järvinen 2003).

Ayurvedic literature describes Arka Kalpana as a liquid dosage form. The procedure resembles the straightforward distillation method used in modern science. Despite not being part of the overall ‘*Panchvidha Kashaya Kalpana*’, this dosage type plays an important role in clinical practice because of its superior bioavailability, lower dosage, greater potency, improved patient compliance, and palatability compared with other drug forms. The device for preparing Arka is known as Arka yantra (Arka Prakash 2019) (Sangeetha et al. 2011). To prepare Arka, Arka Prakasha specifies several water-to-drug ratios for the extraction medium, depending on factors such as the drug's water content, hardness (ranging from soft to hard), and the parts of the drug (ranging from leaves to seeds) Arka Prakasha (Arkaprakāśa 2019). *T. cordifolia* (Willd.) Miers, commonly known as ‘Guduchi’ or ‘Amrita’ in Ayurvedic texts, belongs to the family Menispermaceae and has greenish-yellow, typical flowers found at higher altitudes (Rana et al. 2012). In the Ayurvedic pharmacopoeia, Guduchi is revered as a life-protecting medicinal plant and is widely known by epithets such as Vayastha, Amrita, Jivanti, and Rasayani, reflecting its rejuvenating and vitality-enhancing nature (Joy and Acharya 2022). Recently, the plant has become of great interest to researchers globally owing to its reported medicinal properties, such as anti-diabetic (Acharya et al. 2021), antipyretic, anti-spasmodic, anti-inflammatory, anti-arthritic (Sangeetha et al. 2011), antioxidant, anti-allergic, anti-stress, anti-leprotic, anti-malarial, hepatoprotective, and immunomodulatory (Sangeetha et al. 2011; Chopra, et al. 2011; Bishayi et al. 2002) activities. The stem is abundant in alkaloids (Bishayi et al. 2002), berberine, choline, magnesium, palmatine, tembetarine, tinosporin, sesquiterpenes, and tinocordifolin. jatrorrhizine, tinosporidine, cordifol, cordifelone, giloin, giloinin, glycosides, lactone, and steroids (sitosterols, hydroxyecdysone, makisterone, etc.) (Rai et al. 2024; Saha and Ghosh 2012). By modulating oxidative stress, cytokine balance, and immune regulation, the bioactive constituents tinosporaside, cordifolioside A, magnoflorine, berberine, and giloin contribute to these pharmacological effects (Singh et al. 2023). Pharmacologically, Guduchi exhibits 14 major actions, including rasayana (rejuvenative), balya (strengthening), dipana (digestive stimulant), amahara (detoxifying), and medhya (cognitive enhancer) (Tiwari et al. 2018) actions. Consequently, Guduchi has gained increasing attention in both Ayurvedic pharmaceutics and modern phytopharmacology as a promising source for developing therapeutic formulations, including Guduchi Arka. Guduchi Arka aids in improving Jatharagni (digestive fire) and is beneficial in managing conditions such as Amavata (Rheumatoid arthritis), Vatarakta (Gout), Prameh (increased urine frequency), Raktapitta (hemorrhac disorders)*, *Jvara (fever)*,* and Madhumeha (Diabetes mellitus) (Tripathi 2006).

Thist study aimed to elucidate the pharmacological basis of Guduchi Arka in the management of fever and common cold using in-silico network pharmacology and molecular docking approaches. While fever represents a systemic pyretic response and the common cold is a distinct viral upper respiratory infection, both conditions share a fundamental intersection in host immune-inflammatory signalling, including cytokine cascades and cellular stress responses. Therefore, this computational approach was designed to isolate the overlapping molecular networks shared by these two clinically linked conditions to understand how Guduchi Arka modulates these core pathways. To ensure a scientifically rigorous evaluation, specific analytical techniques were strategically chosen: pharmacopoeial physicochemical testing to establish safety and purity, HPTLC to authenticate the non-volatile baseline of the raw crude drug, and GC–MS to accurately profile the volatile and thermolabile bioactive principles unique to hydrodistilled Arka. This study aims to provide a systems-level understanding of therapeutic potential of Guduchi Arka and support its scientific validation in integrative medicine by identifying specific bioactive components, predicting their targets, and mapping key signalling pathways.

## Materials and methods

### Collection and certification

The crude drug used in this study was obtained from three separate geographical locations: Jhansi (Uttar Pradesh), Bengaluru (Karnataka), and Itanagar (Arunachal Pradesh). These areas were carefully chosen to symbolise different climates, altitudes, and geographical zones in India (Central, Southern, and North-Eastern). This geographical variety was chosen to determine whether environmental variables affect the raw drug and to establish rigorous quality control measures, ensuring that the hydro-distillation process produces a product with a consistent phytochemical profile regardless of the crude drug's origin. The raw drugs were authenticated by Dravyaguna and Pharmacognosy experts at the Central Ayurveda Research Institute (CARI) in Jhansi.

### Macroscopic and microscopic characterzation of raw guduchi drug

**Fig. 1:**
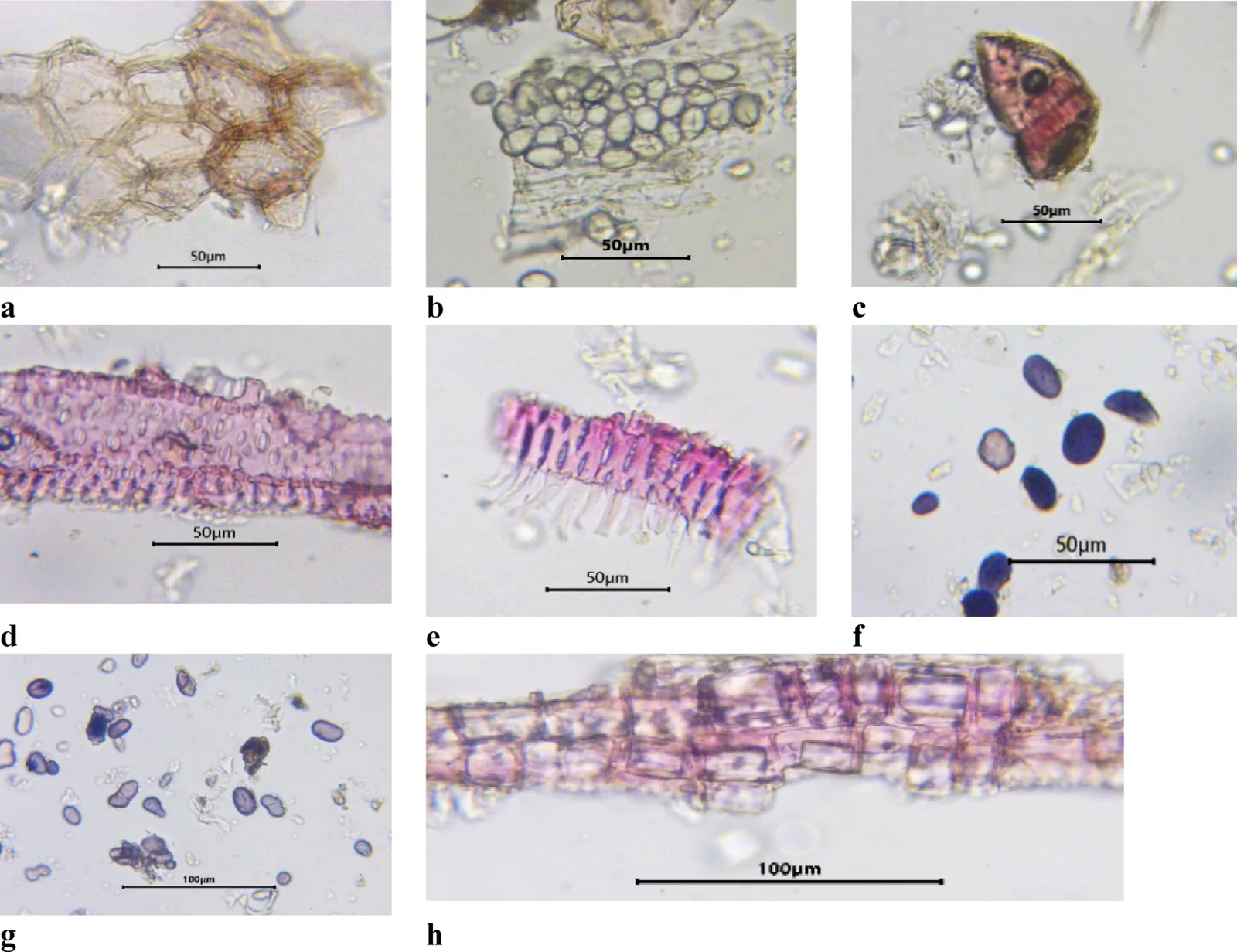
Powder microscopy of Guduchi (Stem) B1(JHA): **a** cork cells in surface view; **b** parenchyma with starch grains **c** lignified stone cells **d** bordered pitted vessels; **e** pitted vesseel; **f** iodine stained simple starch grains; **g** iodine stained simple and compound starch grains; **h** lignified crystal fibres

**Fig. 2:**
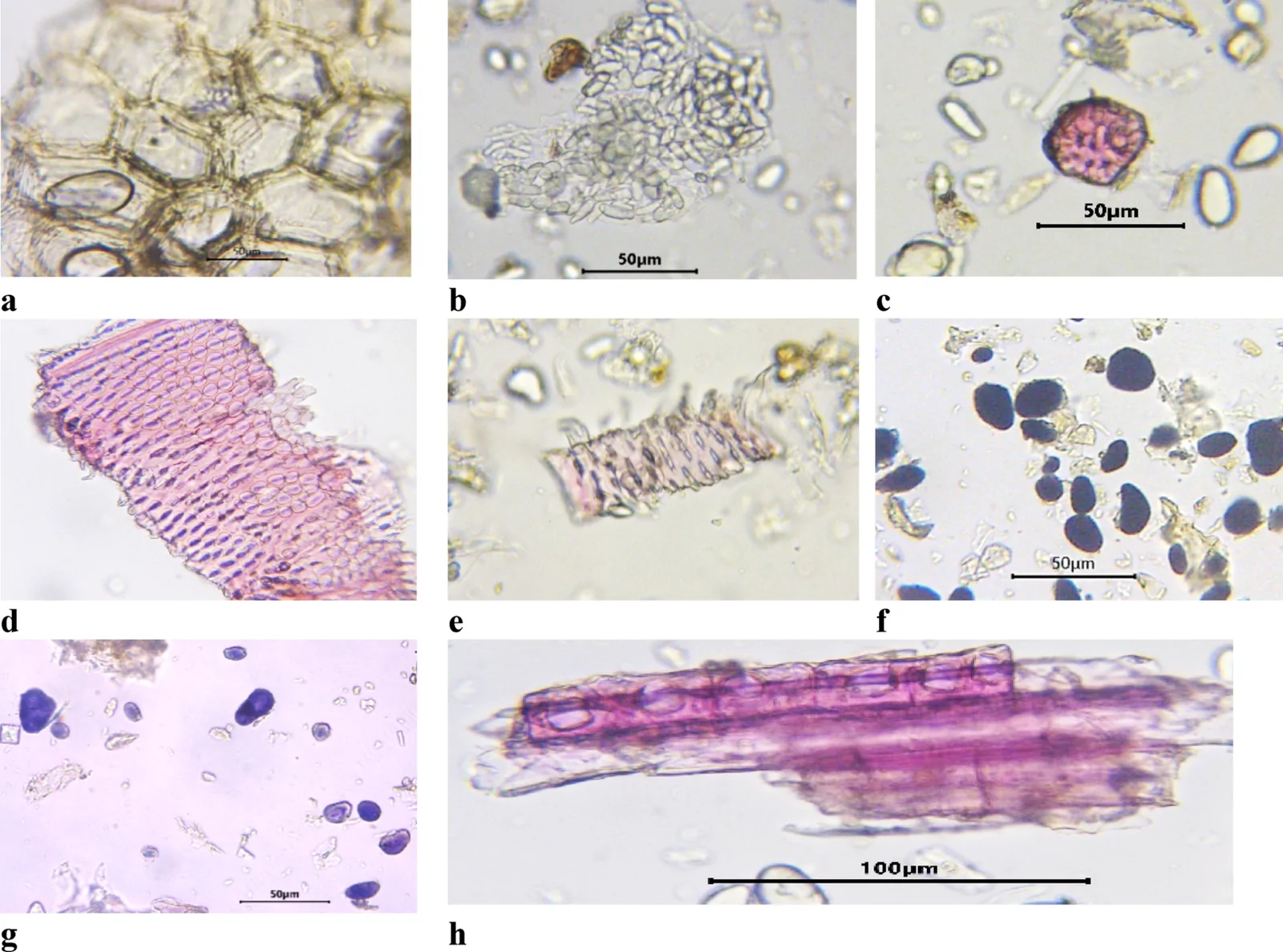
Powder microscopy of Guduchi (Stem) B2(BEN): **a** cork cells in surface view; **b** parenchyma with starch grains **c** lignified stone cells **d** bordered pitted vessels; **e** pitted vesseel; **f** iodine stained simple starch grains; **g** iodine stained simple and compound starch grains; **h** lignified crystal fibres

**Fig. 3:**
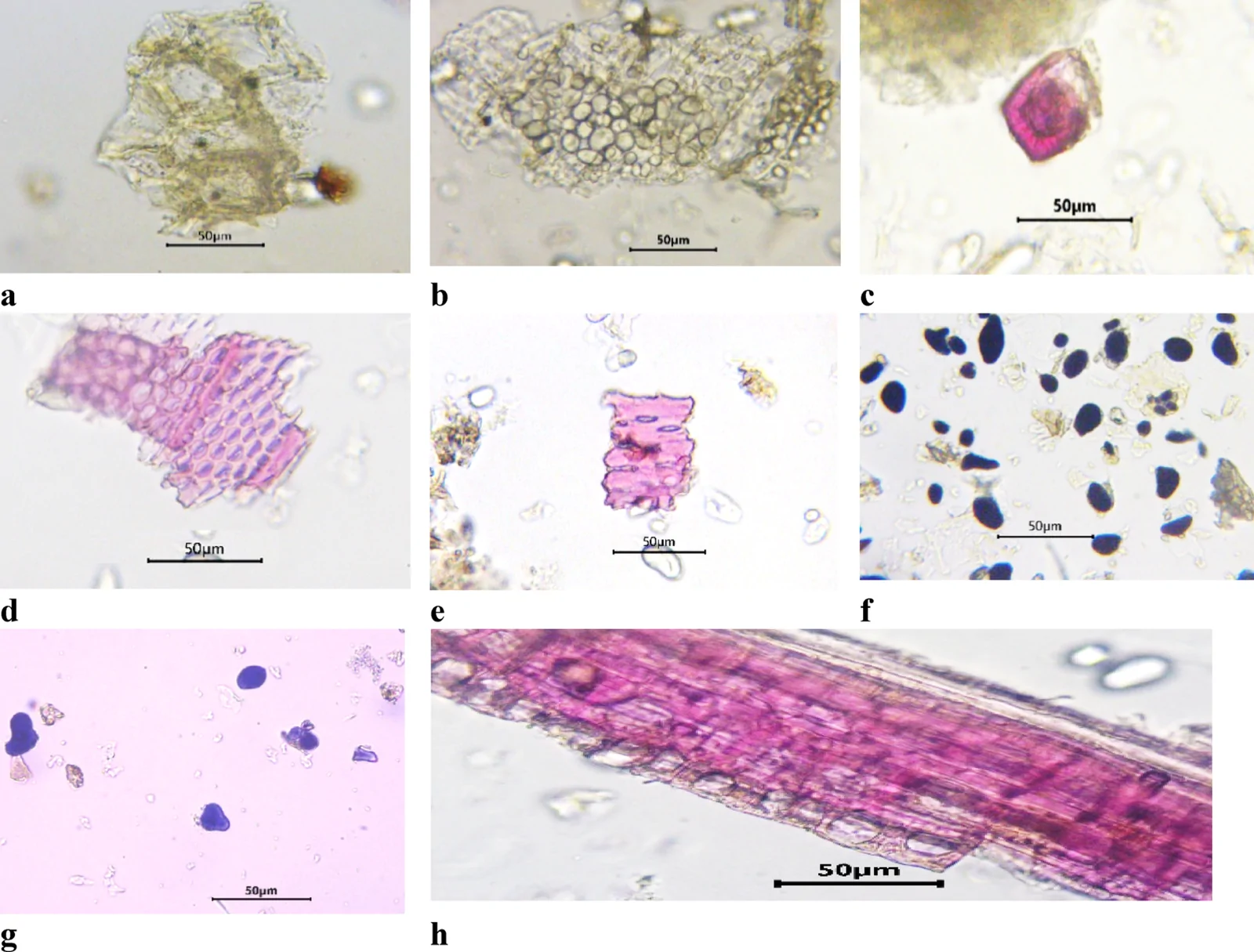
Powder microscopy of Guduchi (Stem) B3(ITA): **a** cork cells in surface view; **b** parenchyma with starch grains **c** lignified stone cells **d** bordered pitted vessels; **e** pitted vesseel; **f** iodine-stained simple starch grains; **g** iodine stained simple and compound starch grains; **h** lignified crystal fibres

*Guduchi* was identified macroscopically based on the length and width, colour, smell, and external characteristics of the sample. To prepare the sample, approximately 1 g of powdered material was placed in a clean beaker. Next, the powder was cleared by heating the mixture over a water bath while adding 10 ml of chloral hydrate (C_2_H_3_Cl_3_O_2_) to the beaker. The cleaned powder was mounted in glycerine on a microscope slide after washing with distilled water. A few milligrams of the cleared powder were treated with iodine in solution for iodine-potassium iodide staining. The stained powder was then mounted in glycerine on a slide. A few milligrams of transparent powder was placed in a watch glass, followed by a few drops of phloroglucinol (C_6_H_3_(OH)_3_) solution, any surplus drained, strong hydrochloric acid, and glycerine mounting on a slide for the phloroglucinol-hydrochloric acid staining procedure. To treat with potassium hydrochloride, a few milligrams of the cleaned powder were boiled in 5 ml of a 5% solution, rinsed with purified water, and then mounted in glycerine. Each slide was examined using a compound microscope set to 10× and 45× magnification, noting and documenting features including cell structures and starch grains in accordance with the Ayurvedic Pharmacopoeia of India (API) guidelines. Data depicted in Table 1 and Figs. 1, 2 and 3 (Minstry of Ayush 2008).

**Table 1:** Organoleptic characters of Raw Guduchi

Organoleptic characters	TC Batch 1 (JHA)	TC Batch 2 (BEN)	TC Batch 3(ITA)
Procured from a geographical location	Jhansi (U.P)	Bengaluru (Karnataka)	Itanagar, (Arunachal Pradesh)
Colour	Brown	Brown	Brown
Odour	Characteristic	Aromatic	Characteristic
Taste	Bitter	Bitter	Bitter
Texture (powdered raw drug)	Coarse with fibrous texture	Coarse with fibrous texture	Coarse with fibrous texture

### Physicochemical characteristics

The physicochemical characteristics of Guduchi, such as pH, Loss on Drying (Minstry of Ayush 2017a), Ash values (Minstry of Ayush 2017), and water- (Minstry of Ayush 2017) and alcohol-soluble extractive values (Minstry of Ayush 2017), were assessed according to the guidelines of the Ayurvedic Pharmacopoeia of India (API). These key analytical criteria were used to determine the fundamental purity of the raw material and to rule out inorganic contamination. To ensure product safety for therapeutic use, heavy metal levels and microbial load were assessed. To establish a baseline fingerprint profile and authenticate the raw Guduchi starting material, HPTLC analysis was performed exclusively on the crude drug. This step was crucial for verifying the chemical consistency across the three geographical batches before distillation. The methanolic extract of the raw drug was applied to silica gel 60 F254 plates, developed using a standardised solvent system, and visualised under UV light at 254 and 366 nm, as well as post-derivatisation. As the final Guduchi Arka is a hydrodistillate containing highly volatile components prone to evaporation during standard HPTLC plate processing (Dane et al. 2026), HPTLC was not used for the Arka. Instead, GC–MS was used to profile the final formulation. The physicochemical analysis data are presented in Tables 2 and 3 (Gandhi et al. 2024).

**Table 2:** Physico-chemical and safety parameters analysis of Guduchi (raw)

S. No	Test(s)	TC Batch 1 Results (n=3)(JHA)	TC Batch 2 Results (n=3)(BEN)	TC Batch 3 Results (n=3)(ITA)	Standard value as per API	Test Method
1.	Loss on Drying (% w/w)	5.66±0.04	5.58±0.03	5.56±0.05		API Part-I, Vol-IX
2.	pH (10% solution)	6.68±0.06	6.61±0.05	6.18±0.07	–	API Part-I, Vol-IX
3.	Water-soluble extractive (% w/w)	19.22±0.12	19.25 ±0.15	19.19±0.1	Not less than 11%	API Part-I, Vol-IX
4.	Alcohol-soluble extractive (% w/w)	7.06±0.04	7.09±0.06	7.07±0.05	Not less than 3%	API Part-I, Vol-IX
5.	Total ash (% w/w)	5.13±0.08	5.16±0.07	5.17±0.09	Not more than 16%	API Part-I, Vol-IX
6.	Acid-insoluble ash (% w/w)	0.62±0.02	0.67±0.03	0.69±0.02	Not more than 3%	API Part-I, Vol-IX
7.	Microbial Load (CFU/g) TBC	1.94×10^6^	1.75×10^6^	2×10^6^	10^7^	API Part-I, Vol-IX
	Microbial Load (CFU/g) TFC	9×10^2^	5×10^2^	9×10^3^	10^5^	API Part-I, Vol-IX
8.	Test for Specific Pathogens (Strain/g) *Escherichia coli, Staphylococcus aureus, Pseudomonas aeruginosa,* and *Salmonella* sp.	Not detected	Not detected	Not detected	<10CFU	API Part-I, Vol-IX
9.	Test for Heavy Metals (ppm) (Pb, Cd, Hg, As)	Below detection limit	Below detection limit	Below detection limit	Pb-10ppm, Cd-0.3ppm, Hg-1ppm, As-1ppm	USP <233>
10	Pesticide residues	Below detection limit	Below detection limit	Below detection limit	–	As per AOAC Official Method 2007.01
11.	Test for Aflatoxins (ppm) (B1, G1, B2, G2)	Below detection limit	Below detection limit	Below detection limit	B1-0.5ppm, G1-0.2ppm, B2-0.1ppm, G20.1ppm	As per Modified AOAC 991.31

**Table 3:** HPTLC analysis of Guduchi (raw)

	TC Batch 1(JHA)	TC Batch 2 (BEN)	TC Batch 3 (ITA)
TLC (R_f_ values) “HPTLC Analysis Report”	**At 254 nm**: 0.014 **At 366 nm**: 0.015,0.201,0.604, 0.698 **Derivatized**: 0.465,0.549,0.899	**At 254 nm**: 0.013 **At 366 nm**: 0.015, 0.204, 0.602, 0.697 **Derivatised**: 0.468, 0.544, 0.896	**At 254 nm**: 0.013 **At 366 nm**: 0.015, 0.204, 0.602, 0.697 **Derivatised**: 0.468, 0.544, 0.896

### Preparation of Guduchi Arka

Guduchi arka was prepared using a pharmacopoeial quality sample of Guduchi. The Guduchi was cleaned, washed, and dried, and 2.5 kg of the selected drug powder was placed in a stainless-steel heating tank along with 25 L of water and soaked overnight. A proper distillation assembly, including a double surface condenser and receiver, was attached. The condenser was connected to a chiller to circulate cool water, and ensure the condensation of the distillate (Arka). The contents of the tank were then heated until boiling commenced, after which the temperature was maintained between 60 and 70 °C. The temperature of the circulating chilled water was precisely maintained at 5–18 °C ± 2 °C using a closed-loop refrigerated recirculating chiller unit. This unit was equipped with a digital Proportional-Integral-Derivative (PID) controller that monitored the coolant return temperature and automatically controlled the cooling system to maintain a consistent temperature gradient across the condenser during the distillation. After approximately 20 min, the Arka began to emerge from the exit. Once a steady flow was established, approximately 5 L of distillate was collected. Distillation was maintained until vapours appeared in the glass receiver, indicating the completion of the procedure. The collected distillate, known as Arka, was then kept or packed in suitable pharmaceutical-grade airtight plastic containers to preserve it from light and moisture (Ancy and Sreekumar 2020).

### Physicochemical and microbial analysis of Arkas of Guduchi

Quality control analysis of Guduchi Arka was performed to determine physicochemical parameters, including pH, specific gravity, and total solid content. Microbial analysis was performed to assess the total viable count, yeast, mould, and pathogen contamination in accordance with standard Ayurvedic pharmacopoeial guidelines to ensure product safety and purity. The physicochemical and microbial analysis data are presented in Table 4 (Minstry of Ayush 2017b).

**Table 4:** Physico-Chemical and Microbial analysis of Arkas of Guduchi

Name of Sample:		GA Batch 1(JHA)	GA Batch 2 (BEN)	GA Batch 3 (ITA)	Test method
S. No	Test(s)	Results (n=3)	Results (n=3)	Results (n=3)	
1.	pH (10% aq. solution)	6.95±0.02	6.36±0.03	6.15±0.02	API Part-I, Vol-IX
2.	Specific gravity	1.00±0.01	0.99±0.01	1.00±0.01	API Part-I, Vol-VI
3.	Boiling Point (°C)	80.0±0.50	85.0±0.40	83.0±0.50	-
4.	Refractive Index (at 40 °C)	1.34±0.001	1.34±0.001	1.34±0.001	API Part-I, Vol-VI
5.	Viscosity (mp at 25 °C)	9.03±0.02	9.07±0.01	9.269±0.018	API Part-II, Vol-I
6.	Essential oil content (%)	0.23±0.02	0.24±0.003	0.24±0.002	API Part-II, Vol-I
7.	Total Acidity	0.92±0.002	0.92±0.002	0.93±0.0014	API Part-II, Vol-I
8.	Test for Heavy Metals (ppm) (Pb, Cd, Hg, As)	Below Detection Limit	Below Detection Limit	Below Detection Limit	USP <233>
9.	Microbial Load (CFU/g) TBC	1×10^2^	1×10^2^	1×10^2^	API Part-I, Vol-IX
10.	Microbial Load (CFU/g) TFC	1×10^2^	1×10^2^	1×10^2^	API Part-I, Vol-IX
11.	Test for Specific Pathogens (Strain/g) *Escherichia coli, Staphylococcus aureus, Pseudomonas aeruginosa,* and *Salmonella* sp.	Not detected	Not detected	Not detected	API Part-I, Vol-IX
12.	Pesticide Residues	Below Detection Limit	Below Detection Limit	Below Detection Limit	As per AOAC Official Method 2007.01
13.	Test for Aflatoxins (ppm) (B1, G1, B2, G2)	Below Detection Limit	Below Detection Limit	Below Detection Limit	As per Modified AOAC 991.31

### GC–MS analysis of Guduchi and Arkas of Guduchi

**Fig. 4:**
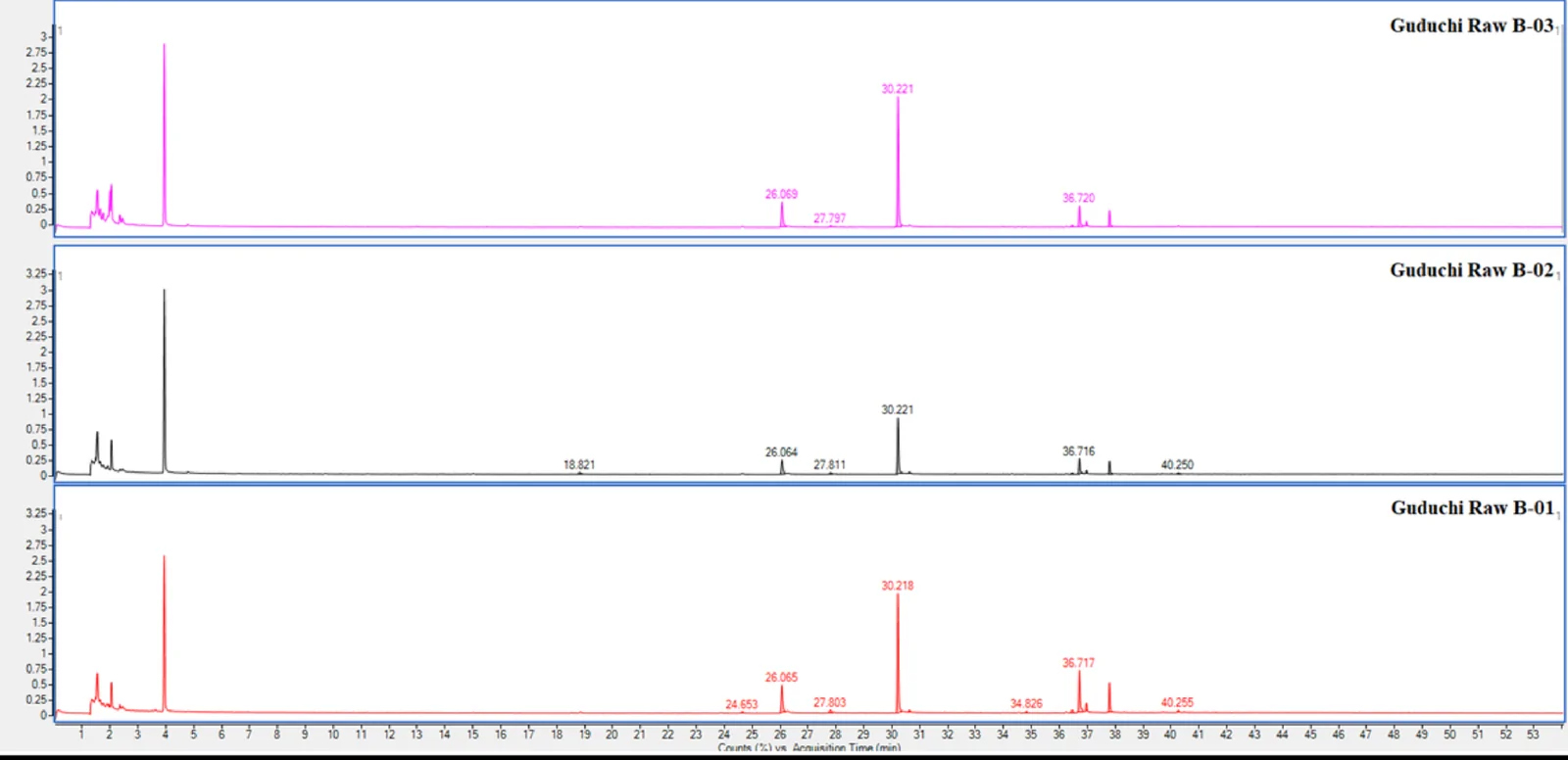
GC–MS analysis of raw Guduchi

Because the Arka hydro-distillation process primarily captures volatile and semi-volatile phytoconstituents that are prone to evaporation during standard TLC/HPTLC plate processing, Gas Chromatography-Mass Spectrometry (GC–MS) was used as the optimal analytical technique for chemical profiling. The Agilent 8890 GC System coupled with 7000D GC/TQ was used in the (Gas Chromatography-Mass Spectrometry) GC–MS/MS analysis of Guduchi arka by employing a combination of two Agilent HP-5MS UI columns (5%-phenyl)-methylpolysiloxane non-polar, 15 m ×250 μm × 0.25 μm). The separation of phytochemicals was carried out using helium as the carrier gas at constant flow rates of 1.2 mL/min in Column I and 1.3 mL/min in Column II. The methanolic extracts, with an injection volume of 1 μL and a split ratio of 5:1, were delivered to the instrument by an auto-sampler (ALS). During the chromatographic run, the initial oven temperature was 60 °C, which was further increased to 280 °C at 5 °C /min (hold time 5 min), and then to 310 °C at 10 °C /min. The conditions of the mass detector were as follows: source temperature 230 °C, mass detector (MSD) transfer line temperature 280 °C, ionisation mode Electron Impact (EI) at 70 eV, scan time 300 ms (0.3 s), in positive and MS2 scan modes. The flow rates of the quench gas He and the collision gas N2 were set at 2.25 mL/min and 1.5 mL/min, respectively. The mass spectra of the major peaks (i.e. major phytochemicals) obtained from the GC–MS chromatograms were compared with those in the GC–MS NIST library. GC–MS analysis of raw Guduchi is presented in Table 5 and Fig. 4 (Bhosale et al. 2024).

**Table 5:** GC–MS analysis of raw Guduchi

S.No	RT (min)	Phytochemicals	Molecular formula	Molecular weight	PubChem CID	% Peak area TC batch 1 (JHA)	% Peak area TC batch 2 (BEN)	% Peak area TC batch 3 (ITA)
1	26.07	Estragole	C_10_H_12_O	148.20	7130	15.5	16.9	17.5
2	27.80	Isolongifolol	C_15_H_26_O	222.37	102,140	01.6	02.2	01.2
3	30.21	Anethole	C_10_H_12_O	148.20	637,563	47.0	48.2	59.6
4	30.63	Thymol	C_10_H_14_O	150.22	6989	00.8	01.5	–
5	34.82	Dalbinol	C_23_H_24_O_7_	412.40	14,227,092	00.9	–	–
6	36.71	Aromandendrene	C_15_H_24_	204.35	91,354	17.7	13.7	11.0
7	36.96	Thujopsene-I3	C_15_H_24_	204.35	28,984	03.2	02.5	02.1
8	37.79	Lupeol	C_30_H_50_O	426.70	259,846	10.8	09.5	07.2
9	40.25	Betulin	C_30_H_50_O_2_	442.70	73,095	00.7	01.3	–

### Network pharmacology study of guduchi phytochemicals

- Identification of phytoconstituents and their targets

Phytochemicals in Guduchi arka were identified using by GC–MS analysis. Phytoconstituents with peak areas greater than 1% were used for target identification. The PubChem database (“PubChem” 2026) provided the 2D structures, PubChem IDs, and canonical SMILES of the phytoconstituents. Phytoconstituents were used to identify putative targets from the Swiss Target Prediction (“SwissTargetPrediction” 2026). In the investigation, only targets related to "Homo sapiens" with a probability score greater than 0.1 were considered for further analysis.

b. Collection of disease-related targets

Targets associated with fever and cold-like symptoms were identified using the phrases "Fever, Cold" and "Homo sapiens" in GeneCards (“GeneCards - The Human Gene Database”. 2026).

c. Common Target Identification

To identify common genes, the intersection of genes between the disease targets and phytoconstituents was generated using Venny 2.1.0 (https://bioinfogp.cnb.csic.es/tools/venny/).

d. Protein–protein interaction

In String 11.0 (“PEAKS Online - Bioinformatics Solutions Inc”. 2026), the common targets for protein–protein interactions between the illness and phytoconstituents are shown. PPI was developed using the lowest interaction threshold of high confidence > 0.7 and humans. Cytoscape 3.10.2 was used to further analyse this portion of the data. A network in Cytoscape 3.10.2 was then created by connecting the common targets and phytoconstituents of interest.

e. Gene Ontology (GO) and Kyoto Encyclopedia of Genes and Genomes (KEGG) analysis

Shiny GO 0.86 was used to predict the numerous biological processes of Gene Ontology** (**GO), and Kyoto Encyclopedia of Genes and Genomes (KEGG) was used for enrichment analysis. The study's common targets, along with the top 20 biological processes and pathways, were identified by selecting fold enrichment and setting the false discovery rate at 0.05. The data are presented in bubble charts showing p-values, fold enrichments, and gene counts. The GO analysis helped clarify many biological processes, cellular components, and molecular roles. Internet technologies can be used to generate insights into the proteins and pathways involved, advancing understanding of the molecular mechanisms by which Guduchi arka reduces fever and cold (Chen et al. 2015).

### Identification of hub genes and molecular docking validation

The top three hub genes were identified based on the highest number of interacting edges (interaction count) in the STRING database protein–protein interaction network. The top three phytoconstituents were selected based on the highest number of direct interactions with these common targets. Molecular docking was performed between these phytoconstituents and hub genes (Mun et al. 2022).

- Target identification and protein preparation

As part of the pre-processing, the 3D crystal structures of the target proteins, namely HSP90AA1 (PDB ID: 5UGB), EGFR (PDB ID: 4BQG), and HSP90AB1 (PDB ID: 6N8Y), were obtained from the Research Collaboratory for Structural Bioinformatics Protein Data Bank. The 3D structures of the proteins were further processed using BIOVIA Discovery Studio Visualizer 2025. The purification procedure involved removing co-crystallised water, ligands, and heteroatoms from the structures, followed by the addition of polar hydrogens to preserve the ionisation state and stability of the proteins.

b. Ligand preparation

The 3D structures of the bioactive molecules, Embelin and Nicolaiodesin-A, were obtained from the PubChem database as Structure Data Files (SDF) (Kim et al. 2021). The compounds were uploaded into the PyRx virtual screening tool (Dallakyan and Olson 2015), and energy minimisation was performed using the Universal Force Field (UFF) via the Open Babel module (O’Boyle et al. 2011) to obtain the most stable conformations. The energy-minimised ligands were subsequently converted to the requisite PDBQT format for docking.

c. In silico molecular docking

Molecular docking was performed using the PyRx program (Dallakyan and Olson 2015), which utilises the AutoDock Vina algorithmic framework (Trott and Olson 2010). The prepared target proteins uploaded to the PyRx program were converted into macromolecule PDBQT files. A grid box was established and centred around the active site of each protein to properly encapsulate the binding pocket. The grid parameters (X, Y, and Z coordinates) were adjusted to allow for unrestricted rotation of the ligands within the active site. The docking process was performed at standard exhaustiveness, and the resulting protein–ligand conformations were ranked by binding affinity (kcal/mol).

d. Docking result visualisation

Post-docking analysis was performed using the BIOVIA Discovery Studio Visualizer. The complex with the lowest binding energy (highest negative value) and optimal root mean square deviation (RMSD) for each ligand–protein pair was selected as the best-docked pose. Two-dimensional (2D) and three-dimensional (3D) interaction maps were generated to evaluate key binding interactions, including hydrogen bonds, hydrophobic contacts, Pi-Pi stacking, and van der Waals forces between the bioactive compounds and the amino acid residues of the target active sites.

## Results

### Macroscopic and microscopic

#### Macroscopic

Macroscopic assessment of three batches of the crude drug obtained from Jhansi (JHA), Bengaluru (BEN), and Itanagar (ITA) revealed a high degree of similarity in physical appearance, despite differences in the places of sourcing. The samples from all three regions had the same brown colour, bitter taste, and coarse, fibre-like texture when powdered. Details comparison depicted in Table 1

#### Powder microscopy

Powder microscopy shows the presence of cork cells in surface view, parenchymatous cells with starch grains, lignified stone cells, bordered pitted vessels, fragments of pitted vesssels, iodine-stained simple and compound starch grains, and lignified crystal fibres. All the observed characteristics of three batches comply with the Ayurvedic Pharmacopoeia of India, Part I, Vol I, Monograph no. 27.

### Physicochemical, safety parameters and HPTLC analysis

#### HPTLC analysis

HPTLC fingerprinting was performed to evaluate the qualitative chemical equivalence of the crude drug batches. HPTLC of the 100 mg/mL-1 methanolic extract (prepared by sonication for 15 min at 25 °C, centrifugation at 10,000 rpm for 5 min, and collection of the supernatant) was performed on pre-coated silica gel 60F254 TLC plates of 0.2 mm thickness (Merck) using the solvent system Toluene: Ethyl Acetate: Formic acid (7:3:0.1). The chromatographic patterns demonstrated highly comparable Rf values across TC batches 1, 2, and 3, confirming that despite geographical variations, the raw starting materials possessed a consistent non-volatile and semi-volatile phytochemical baseline. The HPTLC results for the methanolic extract are shown in Table 3.

## Physico–chemical and microbial analysis of Arkas of Guduchi

### GC–MS analysis of Guduchi Arka

**Fig. 5:**
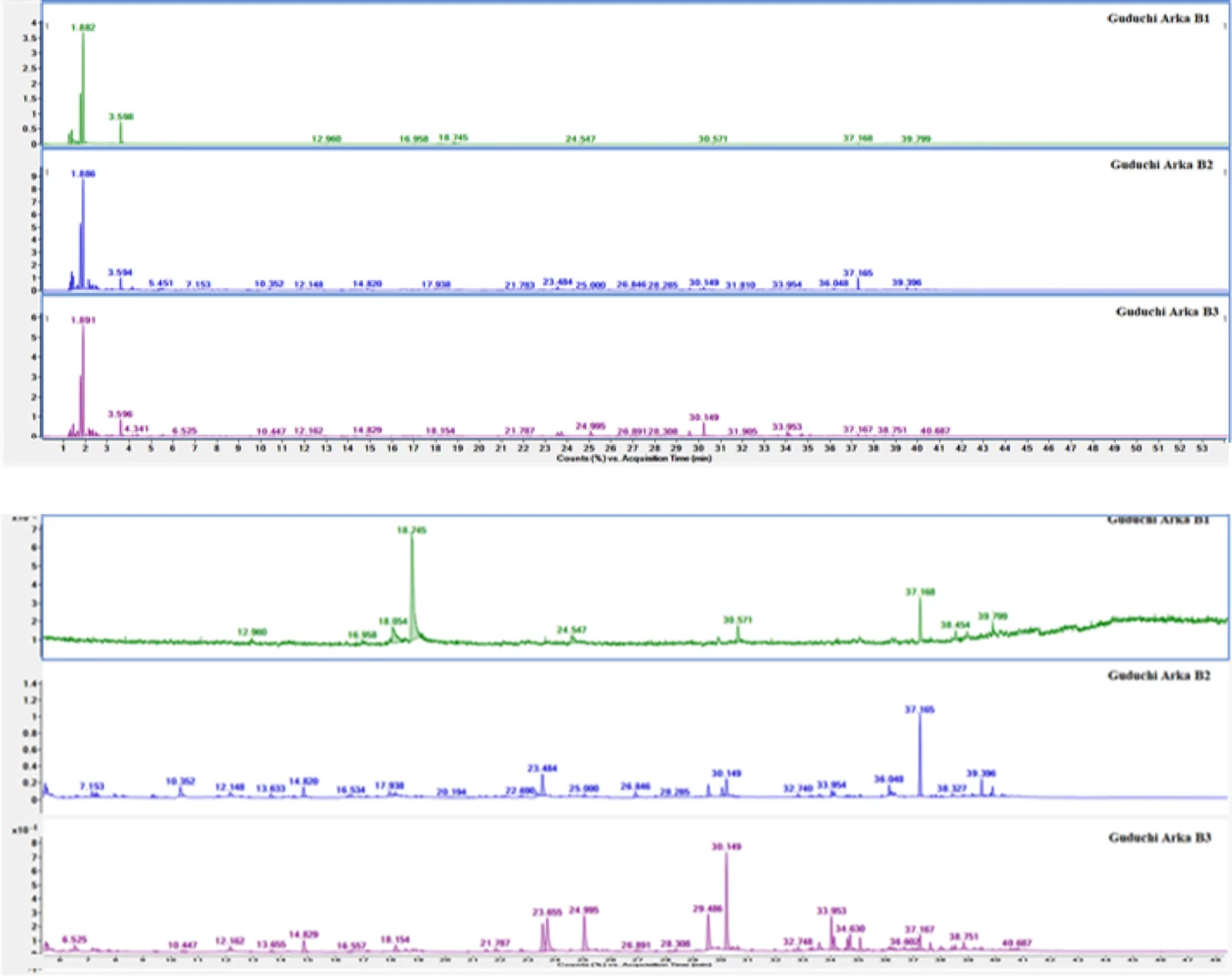
GC–MS chromatograms of Guduchi Arka B1, B2, and B3

The GC–MS chromatograms of Guduchi Arka B1, B2, and B3 are shown in Fig. 5, and the phytochemical analysis is presented in Table 6. Guduchi Arka's GC–MS study showed that all three batches (B1, B2, and B3) contained multiple bioactive phytoconstituents.

**Table 6:** GC–MS analysis of Arkas of Guduchi

S.No	RT (min)	Phytochemicals	Molecular formula	Molecular weight	PubChem CID	% Peak Area GA Batch 1 (JHA)	% Peak Area GA Batch 2 (BEN)	% Peak Area GA Batch 3 (ITA)
1	05.99	α-Cyclogeraniolene	C_10_H_18_	138.25	5,364,405	0.09	0.50	0.07
2	06.09	Traumatic acid	C_12_H_20_O_4_	228.28	5,280,966	0.17	0.37	0.16
3	06.52	1-Eicosanol	C_20_H_42_O	298.55	12,404	1.24	0.33	1.21
4	07.74	Arginine	C_6_H_14_N_4_O_2_	174.20	6322	0.48	0.14	0.32
5	08.39	Isoborneol	C_10_H_18_O	154.25	11,623	0.09	0.78	0.05
6	09.34	Folic Acid	C_19_H_19_N_7_O6	441.4	6037	–	0.39	–
7	14.08	Pinolenic acid	C_18_H_30_O_2_	278.4	5,312,480	–	0.28	–
8	14.22	Fumagillo	C_16_H_26_O_4_	282.38	11,263,051	0.27	0.23	0.17
9	16.55	Cedrene	C_15_H_24_	204.35	410,185	0.53	1.72	0.45
10	16.93	cis-α-Bisabolene	C_15_H_24_	204.35	5,373,003	0.10	0.43	0.06
11	17.72	Verbenol	C_10_H_16_O	152.23	29,015	0.11	–	0.09
12	18.15	α-Campholenal	C_10_H_16_O	152.23	61,050	1.53	1.32	1.49
13	18.33	Coumarine	C_9_H_6_O_2_	146.14	323	–	0.24	–
14	18.53	Cinnamaldehyde, (E)-	C_9_H_8_O	132.16	637,511	0.42	0.31	0.37
15	18.76	trans-α-Bergamotene	C_15_H_24_	204.35	5,308,647	0.37	–	0.29
16	18.89	Ginsenoyne E	C_14_H_20_O_2_	220.31	13,919,421	0.25	0.36	0.20
17	19.10	Peruvinine	C_15_H_22_O_3_	250.33	10,006,240	0.25	0.30	0.21
18	20.04	2,3-Dimethylhydroquinone	C_8_H_10_O_2_	138.16	14,283	–	0.16	–
19	20.79	Columbin	C_20_H_22_O_6_	358.4	119,056	0.14	0.23	0.11
20	22.18	Cyperanic acid	C_15_H_22_O_2_	234.33	13,911,364	0.13	0.30	0.10
21	22.69	Linolenic acid	C_18_H_30_O_2_	278.43	5,280,934	0.80	1.06	0.71
22	23.28	Menthol	C_10_H_20_O	156.27	16,666	–	1.45	–
23	23.65	o-Toluric acid	C_10_H_11_NO_3_	193.20	101,416	9.75	8.25	9.65
24	24.99	Anethole	C_10_H_12_O	148.20	637,563	8.89	0.16	7.62
25	25.00	Longiverbenone	C_15_H_22_O	218.34	588,265	–	1.24	–
26	25.43	Cuparenal	C_15_H_20_O	216.32	13,854,770	0.34	0.43	0.29
27	25.66	Coronarin E	C_20_H_28_O_2_	300.4	10,459,528	0.16	0.18	0.11
28	26.89	Corymbolone	C_15_H_24_O_2_	236.35	11,624,535	0.62	2.40	0.55
29	27.03	Gibberellic acid	C_19_H_22_O_6_	346.4	6466	0.14	–	0.11
30	27.58	β-Nootkatol	C_15_H_24_O	220.35	11,847,668	0.30	0.71	0.21
31	28.01	Ambrosin	C_15_H_18_O_3_	246.30	101,858	–	0.16	–
32	29.17	Pentalenic acid	C_15_H_22_O_2_	234.33	12,304,917	0.19	0.21	0.10
33	29.48	Estragole	C_10_H_12_O	148.20	7130	8.16	3.82	7.65
34	29.85	Geldaramycin	C_29_H_40_N_2_O_9_	560.6	5,288,382	0.33	2.74	0.29
35	30.39	Antibiotic NFAT 133	C_26_H_33_N_3_O_4_S	483.6	73,432,128	0.91	0.90	0.90
36	30.56	trans-Calamenene	C_15_H_22_	202.34	14,210,488	0.68	0.23	0.65
37	32.31	Lovastatin	C_24_H_36_O_5_	404.5	53,232	0.17	–	0.11
38	32.59	Mevastatin	C_23_H_34_O_5_	390.5	53,231	0.36	0.16	0.29
39	32.74	Embelin	C_17_H_26_O_4_	294.4	3218	1.10	1.14	0.90
40	34.43	Arteannuic acid	C_15_H_22_O_2_	234.33	3,032,732	0.30	0.21	0.25
41	34.78	Kadsurin A	C_21_H_24_O_6_	372.4	13,915,159	–	0.40	–
42	35.29	trans-β-Ionone	C_13_H_20_O	192.30	638,014	0.44	–	0.38
43	35.81	Metanephrine	C_10_H_15_NO_3_	197.23	5013	0.45	–	0.38
44	36.12	Betulin	C_30_H_50_O_2_	442.7	73,095	–	0.63	–
45	36.23	Nicolaioidesin A	C_16_H_20_O_3_	260.33	11,575,459	1.23	0.50	1.2
46	36.87	Ergotamine	C_33_H_35_N_5_O_5_	581.7	8223	0.84	0.20	0.74
47	37.16	(-)-Globulol	C_15_H_26_O	222.37	641,460	3.94	20.45	3.86
48	37.90	Paliclavine	C_16_H_20_N_2_O	256.34	119,864	1.95	0.58	1.84
49	38.75	Chamazulene	C_14_H_16_	184.28	10,709	2.54	0.98	2.47
50	38.93	Cadinol T	C_15_H_26_O	222.37	101,037	–	0.23	–
51	39.21	Azafrin	C_27_H_38_O_4_	426.6	5,281,223	–	0.25	–
52	39.40	Oleanolic acid	C_30_H_48_O_3_	456.7	10,494	0.79	4.10	0.61
53	39.79	γ-Eudesmol acetate	C_17_H_28_O_2_	264.4	12,058,444	–	2.84	–
54	40.11	Retinoic acid	C_20_H_28_O_2_	300.4	5,282,379	0.30	0.81	0.24
55	40.53	(E)-Stilbene	C_14_H_12_	180.25	638,088	0.42	0.21	0.25

### Phytochemical composition of *Tinosporia cordifolia* arka and their target identification

We retrieved 10 bioactive phytoconstituents (1-Eicosanol, α-Campholenal, o-Toluric acid, Anethole, Estragole, Embelin, Nicolaioidesin A, (-)-Globulol, Paliclavine, Chamazulene) from the GC–MS analysis of the Guduchi arka, which had a percentage peak area of more than 1%. Subsequently using Swiss target prediction, 360 targets were obtained after removing repetitive genes.

### Target identification of disease

Fever and cold were shown to be disease-related targets, and 4785 targets were found using genecard databases.

### Common targets identification and protein–protein interaction

**Fig. 6:**
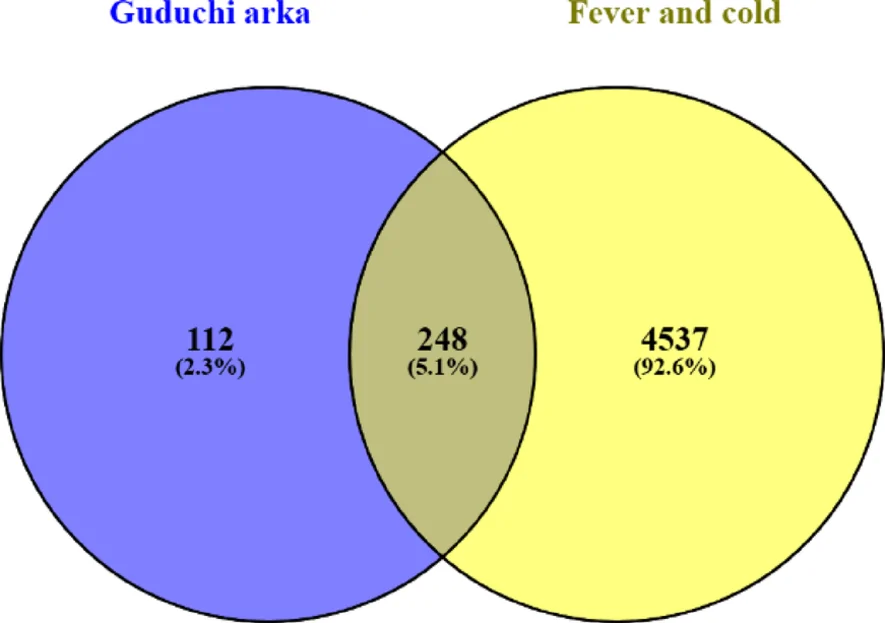
Common targets between the Phytoconstituents and the disease

**Fig. 7:**
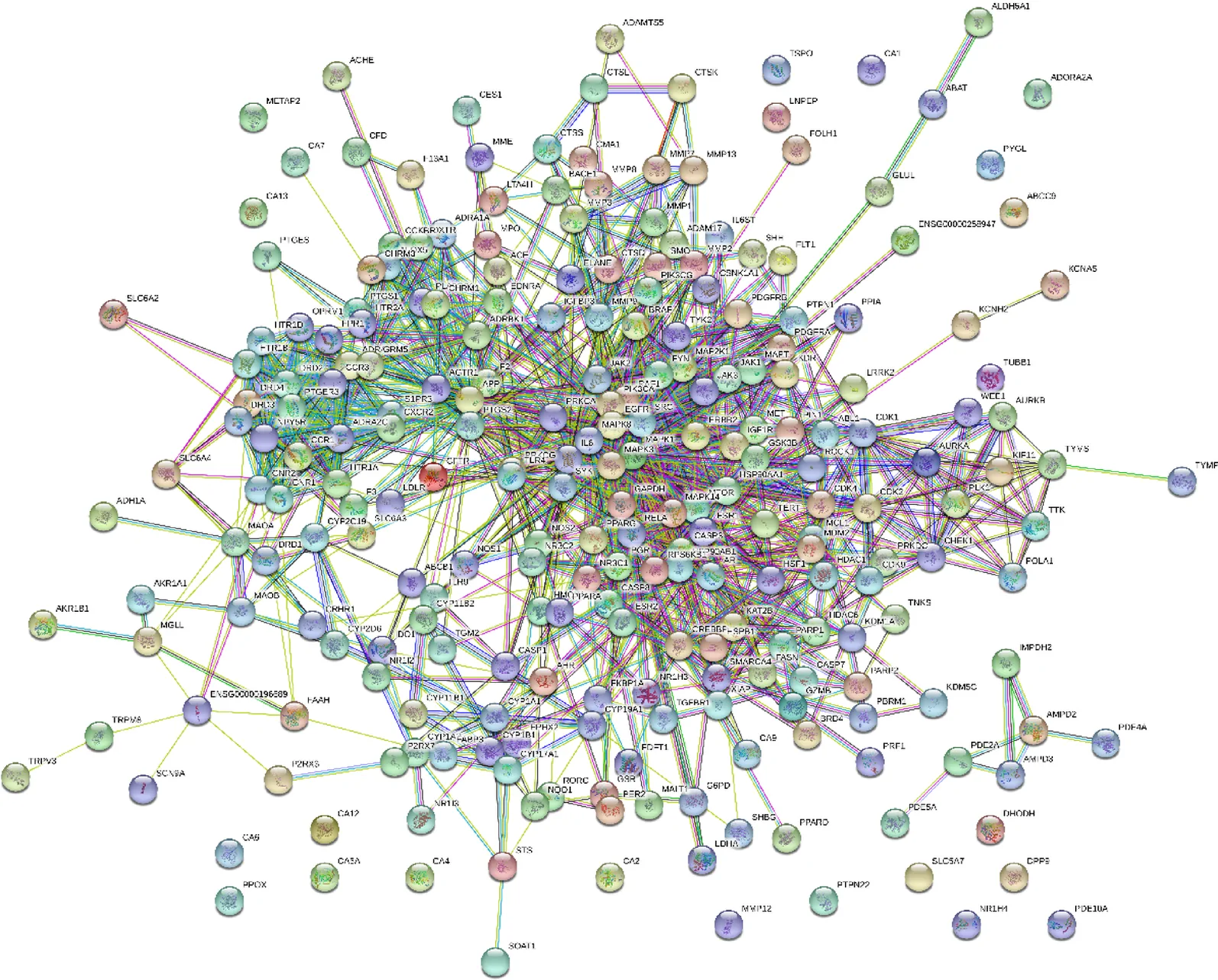
Protein protein interaction between the 248 common proteins

**Fig. 8:**
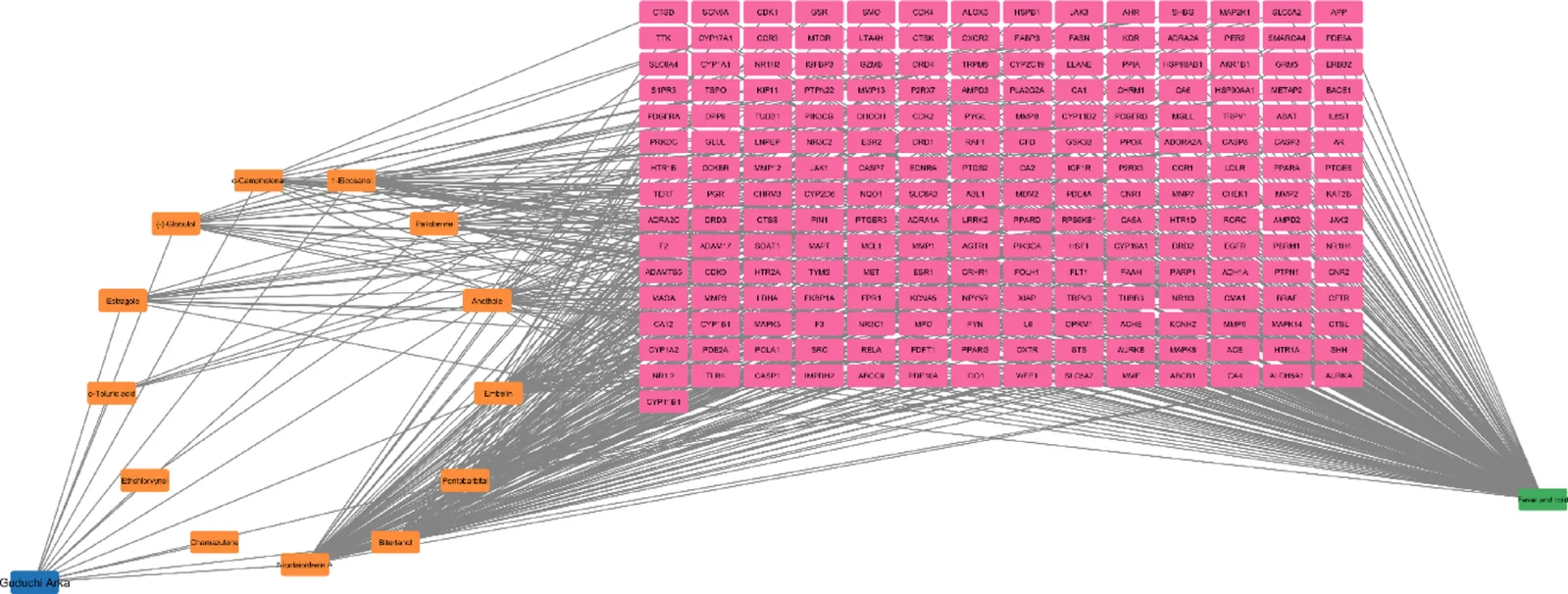
Network between 10 phytoconstituents and 248 common targets

A total of 248 shared targets were between fever and cold phytoconstituent targets (Fig. 6). A total of 1090 edges were identified when these 248 common targets were added to the protein–protein interaction string database (Fig. 7). A high confidence level of 0.70 was established, along with an enrichment p-value of less than 1.0e−16. Cytoscape 3.10.2 was used to build a network linking common targets and phytoconstituents (Fig. 8).

### GO and KEGG enrichment analysis

**Fig. 9:**
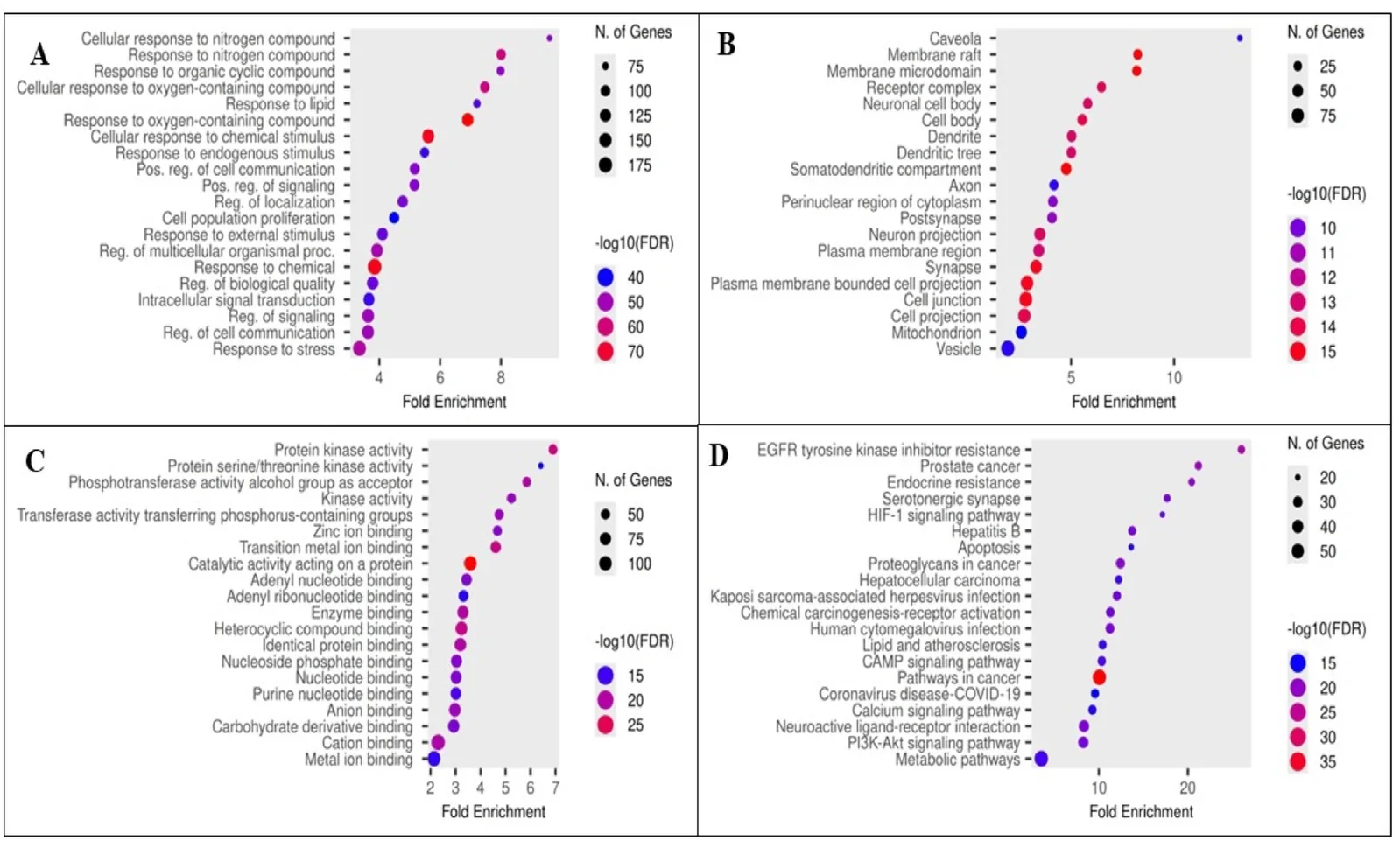
Dot plot of the biological process (**A**), cellular component (**B**), and molecular function (**C**) category terms from GO enrichment analysis and the pathways highly relevant to the treatment effects of Guduchi Arka (**D**) (the X-axis and Y-axis show the fold enrichment and full names of the processes, respectively, and the colour and size of each bubble represent the gene count and –log10FDR, respectively; the subsequent bubble charts are presented similarly)

GO enrichment analysis of common *Tinosporia cordifolia—*fever and cold targets revealed some GO terms associated with the therapeutic effects of *Tinosporia cordifolia* on fever and cold. In Fig. 9A, B, and C, bubble charts representing the top 20 terms from the three categories, biological process, cellular component, and molecular function are shown. These pathways are involved in amelioration of fever and cold by *T. cordifolia,* as shown in Fig. 9D. The major pathways include cancer, metabolic, lipid, and atherosclerosis pathways.

### Hub genes and molecular docking validation

**Fig. 10:**
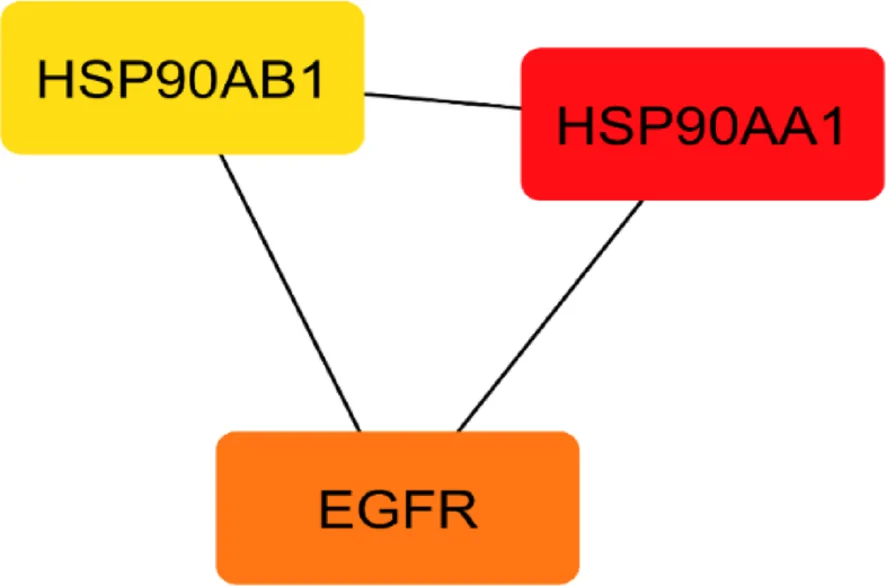
Top 3 hub genes

Based on the highest interaction edge count within the primary PPI network, the top three most interacted genes were identified as the core network hubs. The top three hub genes were HSP90AA1, EGFR, and HSP90AB1 (Fig. 10).

**Fig. 11:**
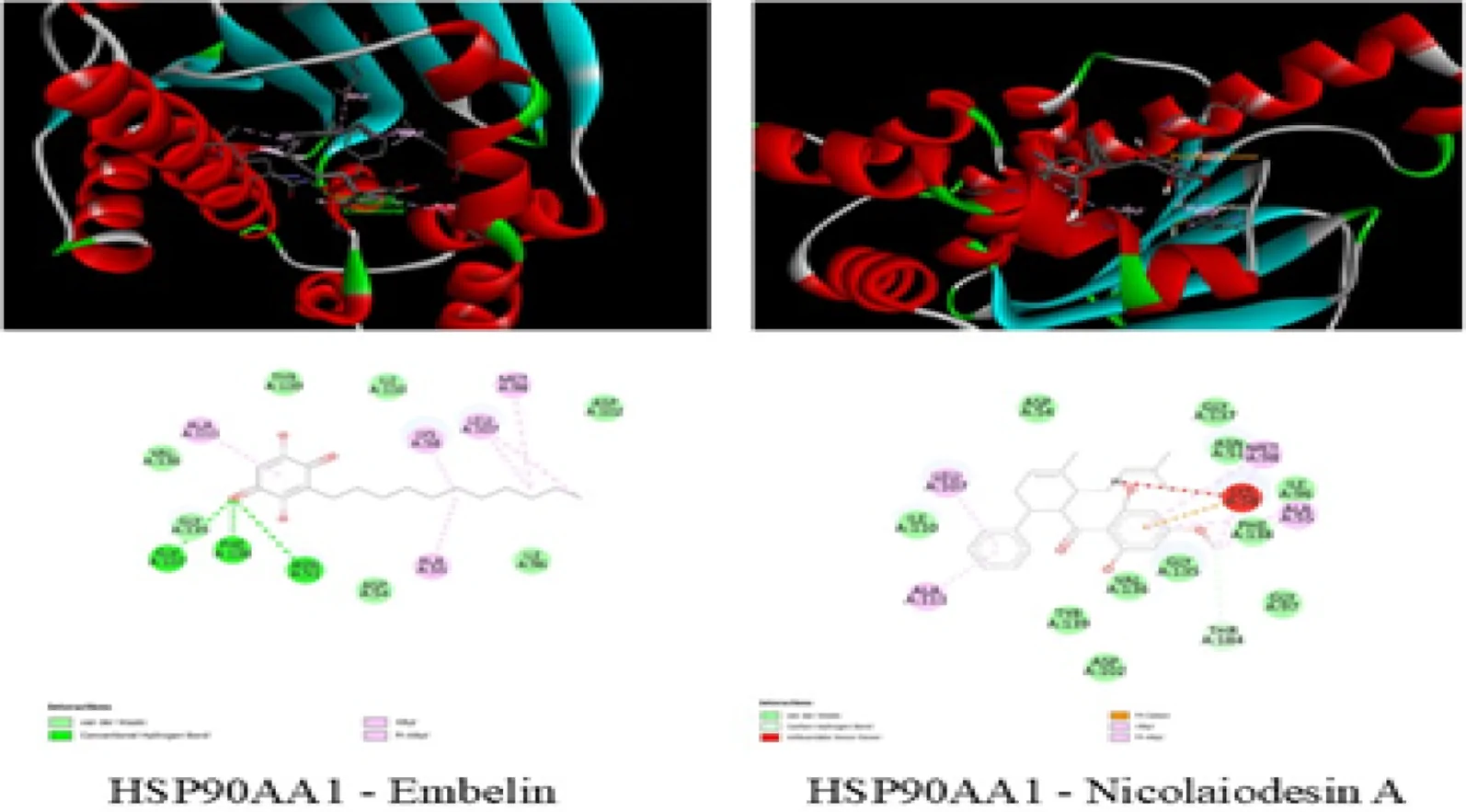
Molecular docking between HSP90AA1 and top phytoconstituents

**Fig. 12:**
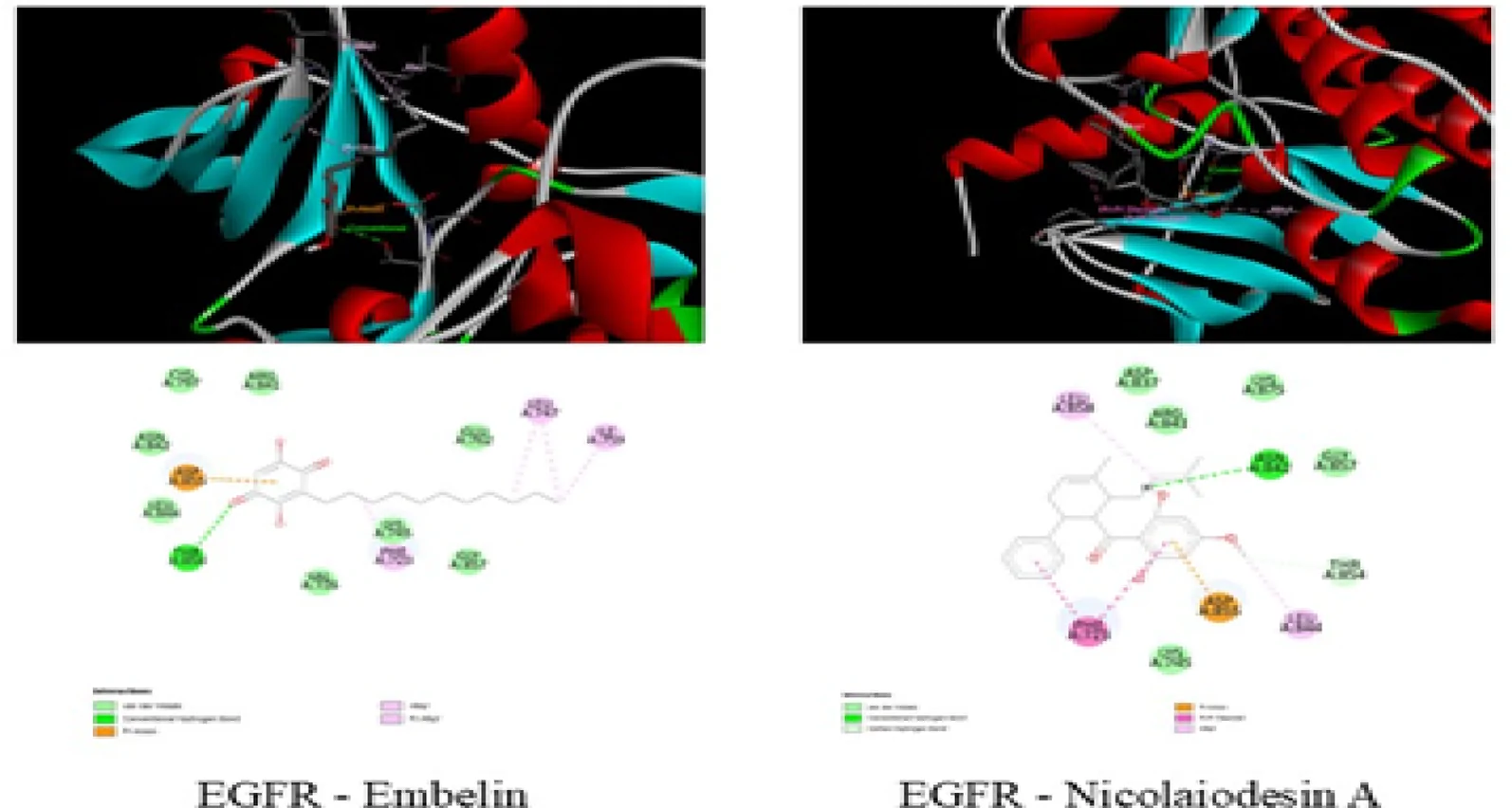
Molecular docking between EGFR and top phytoconstituents

**Fig. 13:**
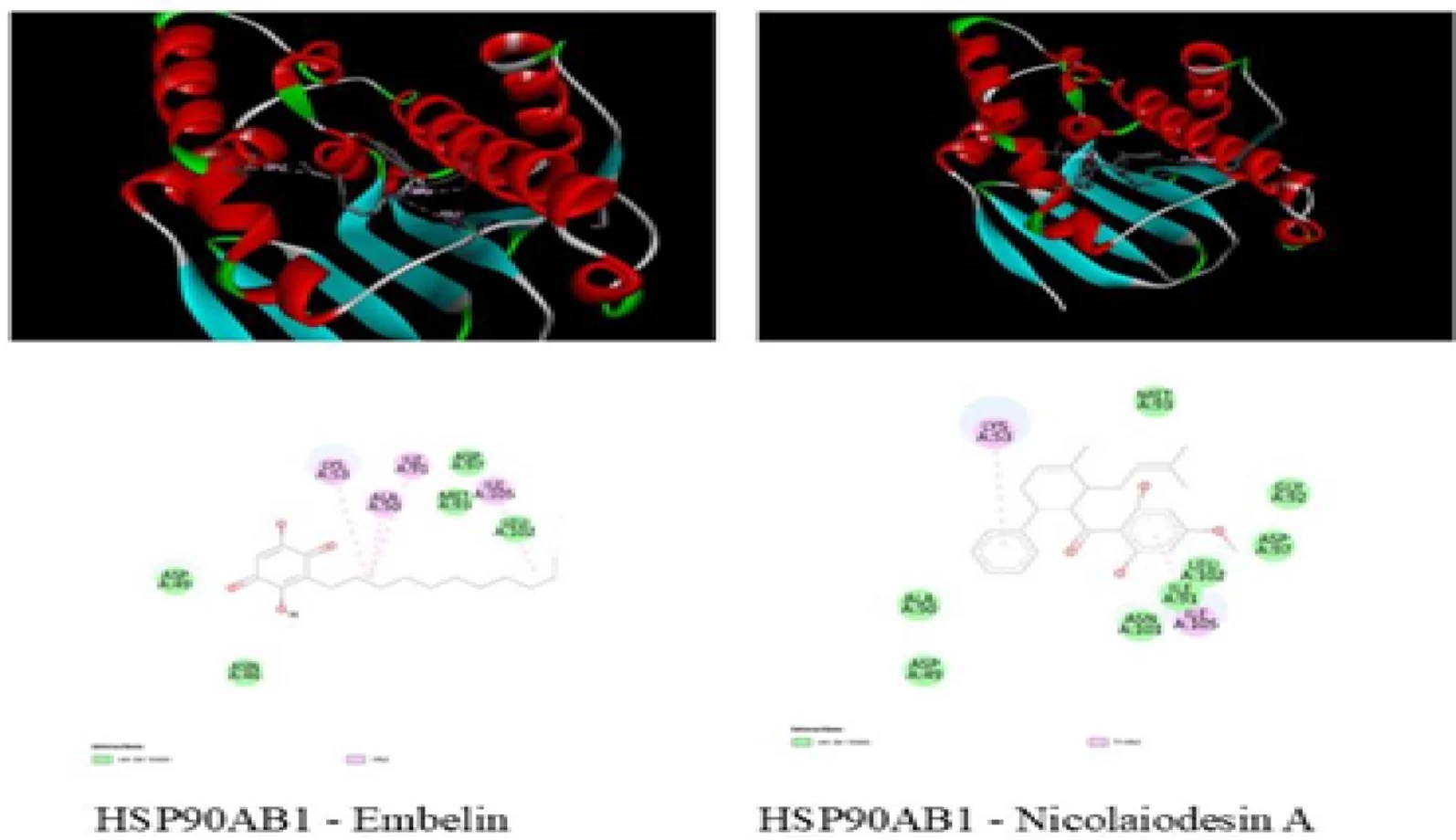
Molecular docking between HSP90AB1 and top phytoconstituents

The top three phytoconstituents were selected based on their interactions with common targets. The top three phytoconstituents were Nicolaiodesin A (77 edges) and Embelin (39 edges). The results of molecular docking revealed that the phytoconstituents successfully oriented within the active pockets of the hub genes, indicating the strong theoretical biological activity of the compounds (Figs. 11, 12, and 13). The docking scores (binding energies) indicated a strong binding affinity, suggesting that Embelin and Nicolaiodesin A stabilise the target proteins (HSP90AA1, EGFR, and HSP90AB1). Although a thorough mapping of specific amino acid interaction networks (such as individual hydrogen bonds and hydrophobic contacts) was outside the scope of our first predictive screening, these extremely favourable binding energies clearly corroborate the network pharmacology predictions. The detailed binding affinities of the molecular docking are depicted in Table 7

**Table 7:** Molecular docking scores of phytoconstituents with various proteins

Bioactive compound/Reference molecule	HSP90AA1 (PDB ID: 5UGB)	EGFR (PDB ID: 4BQG)	HSP90AB1 (PDB ID: 6N8Y)
Embelin	− 6.6	− 5.9	− 5.9
Nicolaiodesin A	− 4.3	− 7.2	− 7.2

## Discussion

### Authentication and quality control of raw material

The quality and conformance of *T. cordifolia* stems were tested according to the Ayurvedic Pharmacopoeia of India. Physicochemical study found moisture content (5.56–5.66%), indicating strong stability against microbial degradation, and a neutral-to-slightly acidic pH (6.61–6.81), ensuring formulation safety. The balanced distribution of water-soluble extractives (19.19–19.25%) and alcohol-soluble extractives (7.06–7.09%) indicates a strong presence of both polar and non-polar elements. In addition, HPTLC fingerprinting established a reliable qualitative baseline, and consistent results obtained under UV and derivatisation conditions verified the uniform presence of non-volatile and semi-volatile secondary metabolites known for their hepatoprotective and immunomodulatory activities.

**Phytochemical Profiling via GC–MS**

Guduchi Arka is a hydro-distillate high in volatile principles, and gas chromatography-mass spectrometry (GC–MS) was used to characterise its chemical complexity. This study verified the effective preservation of important phytochemicals after distillation, including phenylpropanoids (Anethole, and estragole), sesquiterpenoids ((-)-globulol), and quinones (embelin). These findings are consistent with the published literature, in which substances such as anethole, estragole, embelin, and betulin are known for their antioxidant, anti-inflammatory, and hepatoprotective properties.

Importantly, whereas pharmacognosy recognises considerable variability in secondary metabolites based on geographical origin (Desha), our studies show that the Arka Kalpana (hydro-distillation) method serves as a pharmacological equaliser. Despite acquiring raw materials from a variety of habitats, including tropical lowlands and southern plateaus (Jhansi and Bengaluru) and higher elevations (Itanagar), the finished Guduchi Arka exhibited a remarkably consistent phytochemical profile. This indicates that the distillation process successfully standardises volatile medicinal principles, minimises regional variations and ensures consistent clinical effectiveness.

**Mechanistic validation via network pharmacology**

Network pharmacology was used to explore the therapeutic processes of these elements in fever and the common cold. 248 overlapping targets between T. cordifolia phytoconstituents and disease-related genes were identified, demonstrating the multi-component, multi-target paradigm common to Ayurvedic Rasayana treatments.

Protein–protein interaction (PPI) mapping revealed HSP90AA1, EGFR, and HSP90AB1 as major hub genes. These proteins are essential for controlling the inflammatory cascade, immunological responses, and cell viability. The identification of these hubs is consistent with computational research on other immunomodulatory herbs. This convergence shows that these signalling pathways are a key molecular "target set" for Rasayana formulations, thereby altering systemic inflammatory signalling common to both pyrexia and respiratory infections.

**Pathway enrichment and molecular docking**

KEGG and Gene Ontology (GO) enrichment analyses confirmed these targets, identifying PI3K/Akt signalling, metabolic control, and immune-inflammatory cascades as key pathways that reduce oxidative stress and cytokine production during infection. Finally, molecular docking confirmed these relationships, establishing high binding affinities between important phytoconstituents (e.g. Nicolaiodesin A and Embelin) and the active pockets of the identified hub proteins.

In summary, analytical and computational data support the multi-target control of inflammatory pathways as the principal mechanism by which Guduchi Arka exerts its antipyretic and anti-cold properties, providing a scientific foundation for its traditional therapeutic use.

## Conclusion

Guduchi Arka, derived from *T. cordifolia*, was developed in accordance with traditional Ayurvedic guidelines and has been shown to meet critical safety and quality requirements. Chemical profiling identified active substances such as o-Toluric acid, Anethole, Estragole, Embelin, Nicolaioidesin A, and (-)-Globulol, which are known to improve immune function and general health. Computational analysis revealed that compounds such as Embelin and Nicoladaiosin A interact with immune-related targets, including HSP90AA1, EGFR, and HSP90AB1, via key signalling pathways. These findings support the traditional use of Guduchi for fever management and immune enhancement, and offer potential avenues for further research study.

## Data Availability

The datasets used and/or analysed during the current study are available from the corresponding author upon reasonable request.
